# *Serratia marcescens* prosthetic joint infection: two case reports and a review of the literature

**DOI:** 10.1186/s13256-023-04021-w

**Published:** 2023-06-30

**Authors:** Daniel Karczewski, Henrik Bäcker, Octavian Andronic, Angad Bedi, Siegfried Adelhoefer, Maximilian Müllner, Marcos R. Gonzalez

**Affiliations:** 1 Department of Orthopaedic Surgery, Musculoskeletal Oncology Service, Massachusetts General Hospital-Harvard Medical School, 55 Fruit Street, Boston, MA 02114 USA; 2 Department of Orthopaedic Surgery and Traumatology, Charité Berlin, University Hospital, Chariteplatz 1, 10117 Berlin, Germany; 3 Department of Orthopaedics, Balgrist University Hospital, University of Zürich, Forchstrasse 340, 8008 Zurich, Switzerland

**Keywords:** Hip infection, Shoulder infection, Foot infection, Difficult to treat, Gram-negative

## Abstract

**Background:**

Despite some studies on Gram-negative bacteria as difficult to treat pathogens in periprosthetic joint infections, there are no detailed analyses on *Serratia* periprosthetic joint infections. As such, we present two cases of *Serratia* periprosthetic joint infections and summarize all known cases to date in the course of a PRISMA criteria-based systematic review.

**Case presentation:**

Case 1: a 72-year-old Caucasian female with Parkinson’s disease and treated breast cancer developed periprosthetic joint infection caused by *Serratia marcescens* and *Bacillus cereus*, following multiple prior revisions for recurrent dislocations of her total hip arthroplasty. Two-stage exchange was performed, and the patient remained free of *Serratia* periprosthetic joint infection recurrence at 3 years. Case 2: an 82-year-old Caucasian female with diabetes and chronic obstructive pulmonary disease presented with a chronic parapatellar knee fistula after undergoing multiple failed infection treatments at external clinics. After performing two-stage exchange and gastrocnemius flap plastic for combined *Serratia marcescens* and *Proteus mirabilis* periprosthetic joint infection, the patient was released without any signs of infection, but was subsequently lost to follow-up. Review: a total of 12 additional *Serratia* periprosthetic joint infections were identified. Merged with our two cases, the mean age of 14 patients was 66 years and 75% were males. Mean length of antibiotic therapy was 10 weeks with ciprofloxacin most commonly used (50%). Mean follow-up was 23 months. There was a total of four reinfections (29%), including one case of *Serratia* reinfection (7%).

**Conclusions:**

*Serratia* is a rare cause of periprosthetic joint infection affecting elderly with secondary diseases. While the overall reinfection rate was high, the risk of *Serratia* periprosthetic joint infection persistence was low. Treatment failure in patients may be attributable to the host, rather than the *Serratia* periprosthetic joint infection itself, thus challenging current concepts on Gram-negatives as a uniform class of difficult-to-treat pathogens.

*Level of evidence*: Therapeutic level IV

## Background

Periprosthetic joint infections (PJIs) remain a devastating complication of total joint arthroplasty and are expected to increase significantly over the next decades [1, 2]. Gram-negative bacteria resemble rare, yet difficult-to-treat pathogens in PJIs, given resistance to biofilm active antimicrobials, as well as significantly increased rates of treatment failure [3, 4].

Despite its importance, there remains limited knowledge on rare types of bacteria causing PJIs, including the Gram-negative *Serratia marcescens*. Moreover, existing studies include small numbers of patients only, while not differentiating outcome and baseline characteristics among different pathogen classes [5]. A further differentiation of subtypes; however, is essential, as Gram-negative bacteria represent a highly heterogeneous cohort, including pathogens such as *Escherichia coli*, *Pseudomonas aeruginosa*, *Proteus*, *Klebsiella*, or *Morganella* [6], all of them showing different rates of antimicrobial resistances, as well as different reservoirs [7].

*Serratia marcescens*, a rod-shaped facultative anaerobe bacterium is a Gram-negative pathogen that can be found in respiratory and urinary tracts [8]. While *Serratia* is an established cause of catheter-associated bacteremia and urinary tract infections, there is limited knowledge on the ability of *Serratia* to cause PJI [9, 10]. Given limited reports on *Serratia* PJI, as well as the goal to gain a deeper understanding of its role among other Gram-negative PJIs, this study reported two new cases of *Serratia marcescens* PJI and summarized all known cases in the first systematic review to date. We hypothesize that *Serratia marcescens* PJI will demonstrate high failure rates in old and patients with multimorbidities.

## Case reports

**Fig. 1:**
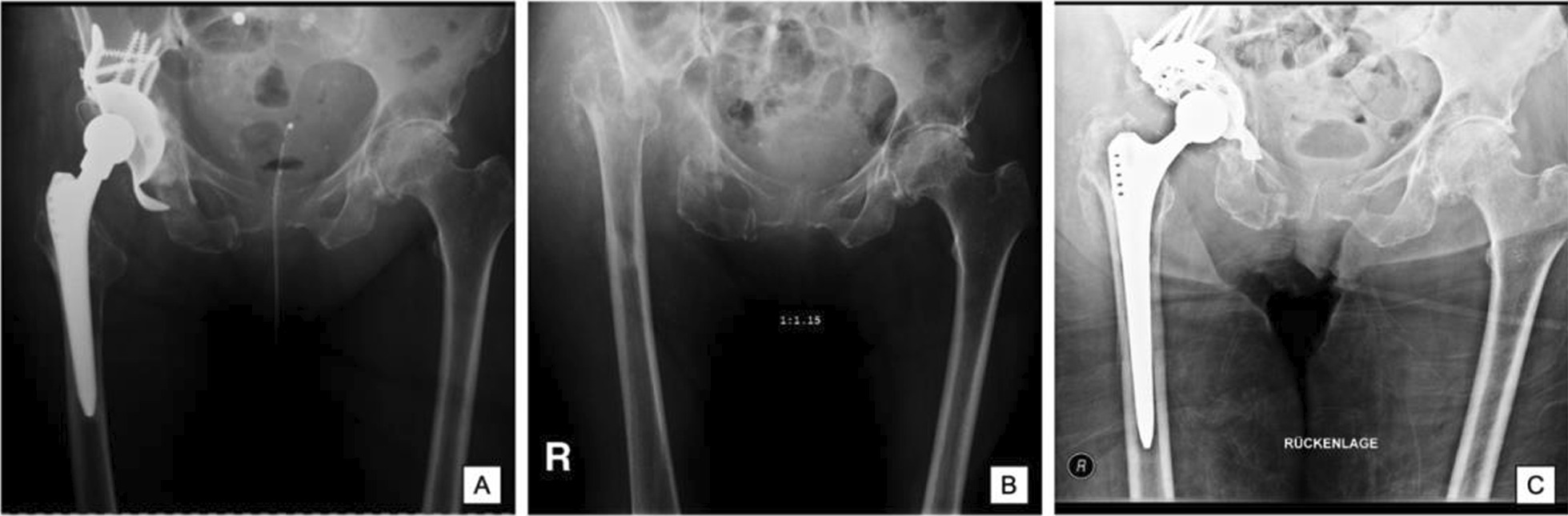
Radiological course of a 72-year-old Caucasian female where **A** pre-first stage, **B** interim phase following two-stage exchange, **C** post-second stage and reimplantation

A 72-year-old Caucasian female underwent a total hip arthroplasty (THA) following a femoral neck fracture at an external community hospital. Comorbidities included Parkinson’s disease, postmamma carcinoma, posthysterectomy, depression, urinary incontinence, congestive heart failure, and rheumatoid arthritis. The family history of the patient was unknown and she was retired following a long-term employment in wholesale commerce. Following recurrent dislocations, open reduction was necessary, and PJI developed thereafter. Debridement, antibiotics, and implant retention (DAIR) is commonly performed following PJI. *Serratia marcescens* and *Bacillus cereus* were identified during the last of several component retention attempts with exchange of head and inlay (Fig. 1A). Since no improvement was noted following multiple external revisions, the patient was multimorbid, and moreover presented with a pancytopenia of unknown origin, and so she was transferred to our university-based interdisciplinary department. The patient complained of pain and she was subfebrile. On arrival, C-reactive protein (CRP) was significantly increased (126 mg/dl). Bone marrow aspiration excluded acute myeloid leukemia (AML). The low cell count was thus attributed to an adverse medication reaction against metronidazole. The patient was diagnosed with *Clostridium difficile* diarrhea at an external community hospital. The diagnosis was made 2 months prior admission to our hospital and she received a 4-week course of metronidazole at the external community hospital. After the patient was cleared by hematologists, we decided to continue with a two-stage exchange (Fig. 1B), given the chronic nature of infection, as well as the disastrous soft tissue conditions. Histopathology obtained during surgery demonstrated signs of osteomyelitis. A total of three tissue samples obtained during surgery remained negative. Care was established in an interdisciplinary setting and with consultations of related medical specialties. Antibiotic therapy was started using meropenem (1000 mg) and vancomycin (1000 mg), each administered three times per day intravenously for 2 weeks, as well as oral clindamycin three times daily (600 mg) and oral ciprofloxacin twice daily (500 mg), each until reimplantation 9 weeks later. No signs of persistent infection were noted during reimplantation, and CRP was normal. Antibiotic therapy was continued with ciprofloxacin (500 mg twice daily) and rifampicin (300 mg twice daily) for a total of 6 weeks. Six days after discharge and 2 weeks after reimplantation, DAIR was performed for persistent wound discharge. Multiple cultures grew *Staphylococcus warneri*, which we considered a contaminant. In our institutional experience, coagulase-negative *Staphylococci*, *Cutibacterium* spp., and *Staphylococcus aureus* were most commonly identified in PJI [11]. In the course of the next 3 years, no further signs of infection developed, although the patient underwent another revision for dislocation at 3 months (Fig. 1C). The patient underwent a 2-month inpatient rehabilitation and was then discharged home. On discharge, she was on metoprolol, digoxin, low molecular weight heparin, levothyroxine, and H2-receptor antagonists.

**Fig. 2:**
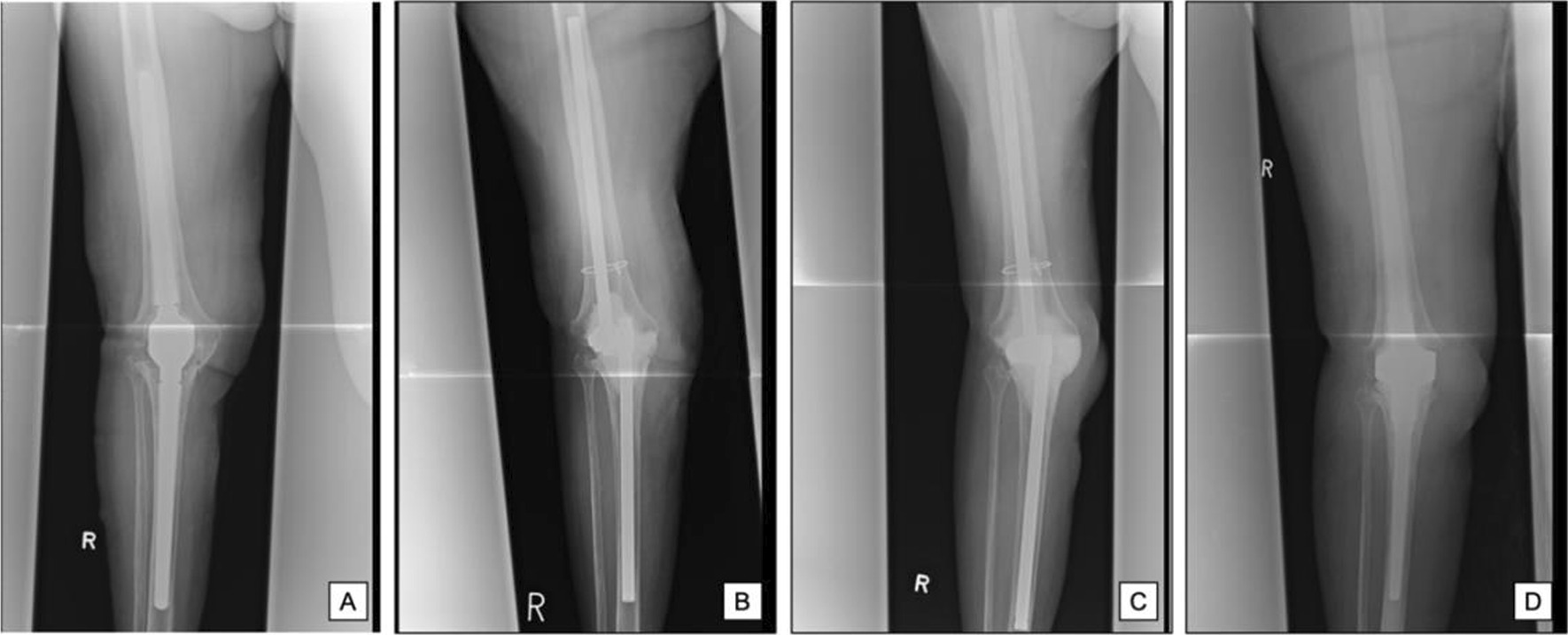
Radiological course of an 82-year-old Caucasian female. **A** pre-first stage, **B** interim phase before spacer exchange, **C** interim phase following spacer exchange, **D** arthodesis

An 82-year-old Caucasian female presented to our outpatient department with pain and subfebrile temperatures. She underwent a substantial number of revisions for infection of the knee at several external institutions for almost a decade. External reports were incomplete. We were able to determine the primary prosthesis implantation to be 15 years previously, and the patient had undergone at least one two-stage exchange for PJI 10 years ago, as well as at least four additional revisions for wound infections. The patient presented to us with a chronic parapatellar knee fistula reaching deep into the knee joint. The CRP was slightly increased at 3.9 mg/dl. The patient could not provide further details on the presence of the fistula. The patient had several comorbidities, namely hypertension, diabetes, chronic obstructive pulmonary disease, epilepsy following meningioma, osteoporosis, and hyperlipidemia. The family history was unknown and she was retired. She received amlodipin, lisinopril, simvastatin, calcium, biphosphonates, and low molecular weight heparin. We decided to proceed with a resection arthroplasty (Fig. 2A). Intraoperative tissue samples grew *Serratia marcescens* and *Proteus mirabilis*, with histopathology confirming the infection. Antibiotic therapy consisted of piperacillin/tazobactam (4500 mg intravenous, three-times daily) and vancomycin (1000 mg intravenous, twice daily) for 1 week each, followed by meropenem (1000 mg intravenous, twice daily). Vancomycin was not administered continuously, although it is hypothesized that a continuous infusion may improve its efficacy. However, a recent review has demonstrated heterogeneous rates of nephrotoxicity [12]. Nine days after prosthesis removal, persistent signs of wound infection, as well as incomplete skin coverage, were noted. As such, spacer exchange using a gentamicin augmented “Arbeitsgemeinschaft für Osteosynthesefragen” (AO) spacer (Fig. 2B, C), as well as an additional skin grafting with a gastrocnemius flap plastic, were performed. Cultures obtained during the procedure remained negative, the patient underwent total knee arthroplasty (TKA) reimplantation 4.5 months later (Fig. 2D), and was prescribed with ciprofloxacin (500 mg, twice daily) and rifampicin (300 mg, twice daily) for 6 weeks thereafter. The patient was discharged home. The patient was released 3 days after reimplantation, but could not be contacted for routine follow-up 3 and 6 months later.

## Patients and methods

The systematic review part of the article was performed on the basis of the Preferred Reporting Items for Systematic Reviews and Meta-Analyses (PRISMA) criteria [13]. PubMed, Web of Science, Ovid Medline, and Cochrane were used as databases. Search criteria were defined as: “*Serratia marcescens* PJI” OR “*Serratia marcescens* joint infection” OR “*Serratia marcescens* arthroplasty infection” OR “*Serratia* PJI” OR “*Serratia* joint infection” OR “*Serratia* arthroplasty infection.” The database search was performed throughout September 2022. Final study inclusion criteria were: (1) *Serratia marcescens* PJI, (2) original studies from 1950 to September 2022, and (3) full English articles. Exclusion criteria were: (1) infection of native joints, (2) infections of osteosynthesis material without arthroplasty, (3) epidemiological studies analyzing the prevalence of *Serratia* infections without any detailed case analysis, and (4) experimental studies. The search was performed by two independent reviewers (DK, MG). Following the removal of duplicated search results, the remaining studies were analyzed, first by title and abstract, and then if considered eligible for inclusion as a full text.

Analyzed parameters included year and country of studies, patient characteristics [age, sex, secondary diseases, Charlson Comorbidity Index (CCI)] [14], joint characteristics (native joint infection, indications for primary arthroplasty, prior revisions), as well as current *Serratia* PJI details (coexisting pathogens, course of symptoms, diagnostical work-up, surgical and antimicrobial treatment). Outcome parameters were length of follow-up, perioperative complications, recurrent infections, and mortality by PJI. Results were descriptively summarized as means for continuous variables, as well as percentages and absolute numbers for categorical variables. The two additional case reports were included in the synthesis of the results.

## Results

**Fig. 3:**
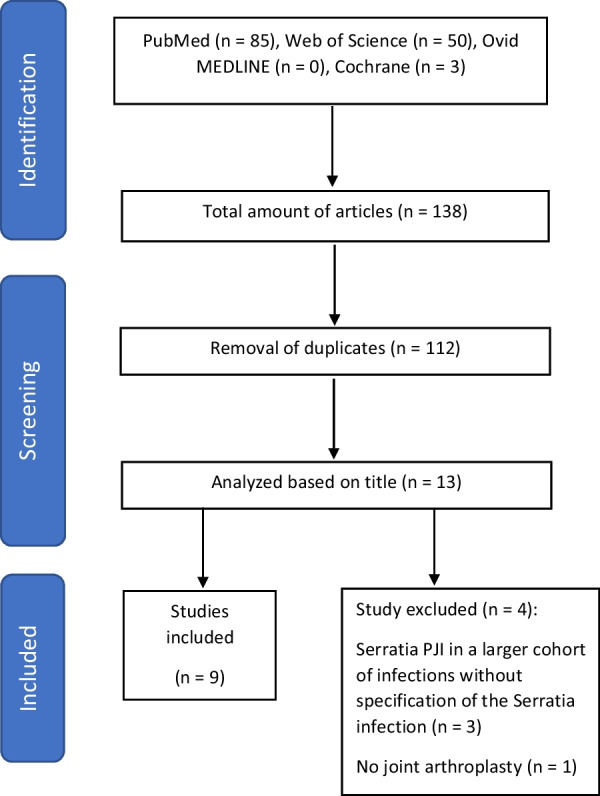
PRISMA flowchart

A total of 138 studies were identified based on PubMed (*n* = 85), Web of Science (*n* = 50), Ovid MEDLINE (*n* = 0), and Cochrane (*n* = 3) searches (Fig. 3). After removal of duplicates, 112 articles remained. Among these, 13 were considered to be possibly eligible for study inclusion, based on their title and abstract. After detailed analysis of main texts, 4 studies were excluded: one as it did not refer to arthroplasty [15] and another three as they analyzed *Serratia marcescens* combined with other pathogens, not allowing for a subanalysis or individual outcome evaluation [16–18].

As such, a total of nine studies with 12 PJIs were included (Table 1) [19–27]. Reports were published between 1993 and 2022 in South Korea, Germany, France, the UK, and the USA (Table 1). Including the two additionally reported cases, there were a total of 14 PJIs, with infections occurring in five total knee arthroplasties (TKAs), four THAs, one reverse shoulder arthroplasty (RSA), one shoulder hemiarthroplasty, one total femoral replacement, one metatarsophalangeal arthroplasty, and one temporomandibular total joint. Mean age was 66 years (range 28–85) with 9 males, 3 females, and 2 unspecified cases of sex. Nine patients had an additional secondary disease, and mean CCI was 1.1 (range 0–4). Hypertension in 5 (36%) and diabetes in 3 cases (21%) were the main secondary diseases identified.

**Table 1:** Summary of baseline characteristics and outcomes among *Serratia marcescens* PJIs

Study	City, country, year	Affected joint	Age	Sex	Secondary diseases	CCI	Native joint infection	Indication for arthroplasty	Prior arthroplasty revision	Coexisting microbe	Onset type	Initial *Serratia* PJI diagnosis	
Lim *et al*. [19]	Seoul, South Korea, 2022	Shoulder, RSA	73	Male	None	0	Together with ORIF material	Posttraumatic nonunion	None	None	16 days, acute	Tissue sample	Intraoperative
Anagnostakos *et al*. [20]	Saarbrücken, Germany, 2021	Hip, THA	68	Male	None	0	NA	NA	NA	None	NA	NA	NA
			69	Male	None	0	NA	NA	Acetabular cup revision	None	NA	NA	NA
		Knee, TKA	71	Male	Hypertension, atrial fibrillation, anxiety disorder	0	NA	NA	NA	None	NA	NA	NA
			85	Male	Hypertension, coronary heart disease, atrial fibrillation	0	NA	NA	NA	None	NA	NA	NA
McKenzie *et al*. [21]	Birmingham, USA, 2017	Temporomandibular total joint	41	Unknown	Chronic ear infection, obesity	0	No	Arthritis	Multiple (Proplast insertion/removal, open arthroplasty)	*Staphylococcus epidermidis*	6 days, acute	Tissue sample	Intraoperative
Cannon *et al*. [22]	Sheffield, UK, 2014	Hip, total femoral replacement	61	Male	Obesity	0	NA	NA	CNS, *Enterobacter cloacae* PJI with periprosthetic fracture	None	Chronic	Tissue sample	Intraoperative
Skedros *et al*. [23]	Salt Lake City, USA, 2014	Shoulder, hemiarthroplasty	58	Male	Diabetes, obesity, hypertension, sleep apnea, COPD, stroke (ataxia), back pain	3	No	Posttraumatic	None	*Candida glabrata*	5 weeks, chronic	Joint aspiration	Preoperative
Mahmoud *et al*. [24]	Liverpool, UK, 2012	Knee, TKA	81	Male	None	0	No	Osteoarthritis, bilateral	None	None	28 days, acute	Tissue sample	Intraoperative
Lefort *et al*. [25]	Paris, France, 2005	Knee, TKA	78	Female	None	0	No	NA	Two-stage exchange for *Staphylococcus aureus*, *Peptococcus* spp., and *Morganella morganii* PJI	None	5 days after prior two-stage exchange, acute	NA	NA
Tannenbaum *et al*. [26]	Ann Arbor, USA, 1997	Hip, THA	28	Unknown	Renal transplant (on prednisone, cyclosporine A)	2	No	Avascular necrosis	None	None	NA	NA	NA
Brink *et al*. [27]	Hines, USA, 1993	Foot, metatarsophalangeal arthroplasty	63	Male	Hypertension, congestive heart failure, cardiomegaly, diabetes, previous smoking and alcohol abuse (quit 2 years before infection)	2	No	Osteoarthritis bilateral, 2 years prior to infection	None	None	2 years, chronic	Tissue sample	Intraoperative
Case report 1	Berlin, Germany, 2022	Hip, THA	72	Female	Parkinson’s disease, prior mamma carcinoma, post hysterectomy, depression, urinary incontinence, congestive heart failure, rheumatoid arthritis	3	No	Posttraumatic	Multiple external revisions for wound infections, coexisting periprosthetic acetabular fracture	*Bacillus cereus*	Chronic, multiple months	Tissue sample (external)	Intraoperative (external)
Case report 2	Berlin, Germany, 2022	Knee, TKA	82	Female	Hypertension, diabetes, COPD, epilepsy following meningioma, osteoporosis, hyperlipidemia	4	No	Unknown, 15 years prior to PJI	Multiple prior external revisions, including two-stage exchange	*Proteus mirabilis*	Chronic, years	Tissue sample	Intraoperative

Study	CRP	ESR	Leading symptom	Surgery for *Serratia* PJI	Antibiotics for *Serratia* PJI	Perioperative complications	Outcome of initial treatment	Subsequent re-revision	Prosthesis *in situ* at last FU	Follow-up	Death by PJI
Lim *et al*. [19]	3.1 mg/dl	NA	Wound dehiscence, purulence	DAIR	Ciprofloxacin, 6 months	None	No clinical signs of reinfection	None	Yes	48 months	No
Anagnostakos *et al*. [20]	NA	NA	NA	DAIR	Rifampicin, meropenem, ciprofloxacin	NA	No clinical signs of reinfection	None	Yes	34 months	No
	NA	NA	NA			NA	No clinical signs of reinfection	None	Yes	36 months	No
	NA	NA	NA	Two-stage exchange	Ciprofloxacin	NA	No clinical signs of reinfection	None	Yes	58 months	No
	NA	NA	NA			NA	Reinfection	NA	NA	6 months	NA
McKenzie *et al*. [21]	NA	NA	Ear pain, otitis externa	Two-stage exchange	NA	None	No clinical signs of reinfection	None	Yes	9 months (after removal)	No
Cannon *et al*. [22]	NA	NA	Dislocated femoral replacement	Open reduction	Ciprofloxacin	None	Reinfection with *Serratia*	Two-stage exchange, spacer	Yes	18 months	No
Skedros *et al*. [23]	5 mg/dl	40 mm/hour	Erythema, fistula	Two-stage exchange to RSA	Piperacillin/tazobactam 4.5 g intravenous/8 hours, micafungin 150 mg intravenous/day, 6 weeks	Fall on shoulder, feeling of instability	Reinfection with *Candida glabrata*	Resection arthroplasty, oral suppression	No	18 months	No
Mahmoud *et al*. [24]	3.8 mg/l	67 mm/hour	Joint hot, swollen painful	DAIR	Meropenem, 3 weeks intravenous, then ciprofloxacin 3 weeks oral	None	No clinical sings of reinfection	None	Yes	8 months	No
Lefort *et al*. [25]	NA	NA	Local and general inflammatory symptoms	Resection	Imipenem (1 g three-times daily), Amikacin (15 mg/kg/d) each for 6 months; Imipenem replaced by Meropenem (2 g t.i.d.) for 6 months, in combination with amikacin for 2 weeks	None	No clinical signs of reinfection	None	No	12 months	No
Tannenbaum *et al*. [26]	NA	NA	NA	Resection	NA	NA	No clinical signs of reinfection	None	No	15 years (since THA, not used for calculation)	No
Brink *et al*. [27]	NA	25 mm/hour	Persistent edema, erythema, superficial ulcer, white exudate	Resection	Cefotetan intravenous, 6 weeks	None	No clinical signs of reinfection	None	No	17 months	No
Case report 1	126 mg/dl	NA	Persistent wound secretion, fever	Two-stage exchange	Meropenem 1 g/50, vancomycin 1 g/100 intravenous each 1–1–1 for 2 weeks, clindamycin 1–1–1 600 mg per os, Ciprofloxacin 1–0–1 500 mg per os until reimplantation, then ciprofloxacin 500 mg 1–0-1 and rifampicin 300 mg 1–0–1, 6 weeks each	Acute kidney injury, atrial fibrillation, delirium, respiratory insufficiency	Reinfection with *Staphylococcus warnerii*	DAIR	Yes	36 months	No
Case report 2	3.9 mg/dl	NA	Fistula parapatellar, chronic	Three-stage exchange	Tazobactam 4.5 g intravenous 1–1–1 and vancomycin 1 g intravenous 1–0–1, each 1 week, then meropenem 1 g intravenous 1–0-1 until reimplantation, then 6 weeks ciprofloxacin 500 mg 1–0–1, rifampicin 300 mg 1–0–1	Asystole/CPR (dislocated intubation tube), hypotonia	No clinical signs of reinfection	None	Yes	3 days	No

One patient had an infected native shoulder joint together with infected osteosynthesis material prior to undergoing reverse shoulder arthroplasty. In addition, the second shoulder and newly reported hip case underwent arthroplasty for posttraumatic joint damage. Moreover, one TKA and the only foot implant, as well as the only temporomandibular total joint, were performed for osteoarthritis. In addition, one THA was performed for avascular necrosis. The remaining cases did not include the precise indication for primary implantation. Six arthroplasties were revised prior to the current *Serratia* PJI, including four for a non-*Serratia* infection.

*Serratia marcescens* was identified through a preoperative joint aspiration in one case, and through intraoperatively obtained tissue samples in all other cases. Four cases showed presence of a coexisting pathogen (*Candida glabrata*, *Bacillus cereus*, *Proteus mirabilis*, *Staphylococcus epidermidis*). A chronic symptom onset was described in five PJIs, whereas four had an acute onset, and the others did not give details about the course of infection. Mean preoperative CRP was 35.2 mg/l and mean erythrocyte sedimentation rate (ESR) was 44 mm/hour.

Classical signs of infection were present in all but one case, in which *Serratia* was identified following open reduction of a dislocated total femoral replacement. The remaining cases were treated with two-stage exchange in five cases, DAIR in four cases, permanent resection arthroplasty in three cases, and three-stage exchange in one patient. Mean length of antibiotic therapy was 10 weeks (range 6 weeks to 6 months). Ciprofloxacin (50%), meropenem (36%), and rifampicin (21%) were the most common antibiotics used. At a mean follow-up of 23 months (range 0.1–58 months), four recurrent PJIs occurred (29%), including one case of *Serratia* reinfection (7%). Four patients had no prosthesis reimplantation at last follow-up, and no patient died as a consequence of the *Serratia* PJI.

## Discussion

*Serratia marcescens* occurs in less than 1% of all PJIs [18]. Importantly, it is part of the group of Gram-negative PJIs that has increasingly being shifted to the center of attention, given poor outcome reports [5, 6]. As Gram-negative PJIs resemble a serious burden to patients, we believed a more detailed subanalysis of *Serratia* PJI to be necessary, and reported two new PJIs combined with the first systematic review to date. Our results demonstrated that one in three patients revised for *Serratia* PJI developed a recurrent infection at a mean follow-up of less than 2 years. Of these, only one case of *Serratia* recurrence was noted.

Knowledge on epidemiological characteristics is essential, as certain pathogens are known to be attributable to certain risk groups [28, 29]. Our cohort demonstrated more than 60% of patients to be affected by a secondary disease. Of note, one in two patients had at least one established risk factor for developing PJI, including obesity, diabetes, rheumatoid arthritis, and immunosuppressive medication [30–32]. Moreover, one in four joints had a trauma in the past, and nearly half were revised before, further increasing the risk of developing infection [33, 34]. Finally, the mean age of our patients was high, and as such in line with previous reports on Gram-negative pathogens [5].

There remains limited knowledge on clinical and diagnostical characteristics of *Serratia marcescens* PJI. We identified reports on infections in all major joints, as well as foot and temporomandibular joint, with the knee being most commonly affected (36%), followed by the hip (33%). Of note, previous investigations found that Gram-negative bacteria more frequently affects the hip, possibly due to the proximity to the gastrointestinal tract [35]. *Serratia marcescens* has not been previously described as a classical contaminant, unlike other pathogens, such as *Cutibacterium* [36]. This is also reflected in an acute symptom onset in nearly half of all patients, low rates of polymicrobial cases, as well as significantly increased CRP and ESR. Importantly, three out of four coexisting pathogens were of atypical nature, including *Bacillus* and *Candida*, possibly reflecting the high-risk cohort described earlier [1, 37].

The selection of an adequate surgical strategy is essential. Current guidelines suggest chronic infections with completed biofilm formation or cases of poor soft tissue condition to undergo a complete prosthesis removal and/or exchange, with DAIR being an option in acute cases [38, 39]. Of note, these guidelines represent the treatments performed in the included patients [11, 40]. PJI eradication can only be achieved by combining an adequate surgical strategy with an adequate antimicrobial therapy [41, 42]. The mean length of 10 weeks of antibiotic therapy identified in our report falls in line with previous investigations on Gram-negative PJIs [35, 43]. Moreover, the majority of cases were treated with ciprofloxacin (50%) and meropenem (36%). This is important, as fluroquinolone-resistant Gram-negative bacteria are considered an additional therapeutical challenge, given limited options in other biofilm active antimicrobials [4]. Although no detailed resistance profiles were available in the majority of included studies, the use of ciprofloxacin in half of all cases indicates a low rate of fluroquinolone-resistant *Serratia* PJIs.

Reinfection rates in the cohort resulting from our systematic review were high with 29% developing a recurrent infection at a mean follow-up of less than 2 years. We believe this devastating outcome to be caused by a number of factors, including a substantial number of secondary diseases in affected patients, old age, as well as a high rate of previous revisions. Importantly, only one patient had a reinfection caused by *Serratia*. This is essential, as it may indicate that *Serratia* PJIs occur in high-risk patients, but can successfully be eradicated by an adequate therapy. This is further supported by one immunocompromised patient who was treated successfully without signs of reinfection at a follow-up of 12 months [26]. As such, reinfections resemble the general risk of these patients, rather than the *Serratia* pathogen itself. This also falls in line with one previous investigation analyzing PJI recurrence by the same pathogen. The authors identified that less than one third of all recurrent infections were by the same pathogen, and *Staphylococci* were the only bacteria class with a statistically significant risk of persistence [44].

This investigation had a number of limitations. Foremost, we report a small number of 14 joints only. Moreover, infections were identified in five different joints, with different surgical strategies used over a time period of more than three decades, limiting overall consistency and comparability. This problem is further increased as PJI was not clearly defined in studies, specifically concerning the number of positive tissue samples obtained during surgery. Finally, our results represent short-term outcomes only.

## Conclusion

*Serratia* PJI is a rare complication that has been described in all major joints, and tends to primarily affect elderly with significant secondary diseases. Although patients are at high short-term risk of reinfection by a different pathogen, there has only been one case of *Serratia* PJI recurrence described to date. Current concepts on Gram-negative bacteria as a uniform class of difficult-to-treat pathogens must be viewed critically, as our results indicate treatment failure to be attributable to the host, rather than the pathogen.

## Data Availability

Made available upon request.
